# From Tea Catechins to Microbial Metabolites: Emerging Mechanisms for Modulating Gut-Origin α-Synuclein Seeding in Parkinson’s Disease

**DOI:** 10.3390/nu18203304

**Published:** 2026-10-09

**Authors:** Ali Rehman, Lili Tang

**Affiliations:** Department of Environmental Health Sciences, University of Georgia, Athens, GA 30605, USA; ali.rehman@uga.edu

**Keywords:** tea catechins, epigallocatechin-3-gallate, microbial metabolites, α-synuclein seeding, cross-seeding, curli, gut microbiota, gut-origin α-synuclein pathology, body-first Parkinson’s disease

## Abstract

Parkinson’s disease (PD) is increasingly recognized as a heterogeneous neurodegenerative disorder with multiple clinical and pathological trajectories. In a subset of patients, α-synuclein pathology may begin in the gut and subsequently spread to the brain, a pathway most relevant to the proposed body-first subtype of PD. Tea catechins, particularly epigallocatechin-3-gallate (EGCG), can influence α-synuclein aggregation, oxidative stress, inflammatory signaling, and gut microbial ecology. Because their systemic bioavailability is limited, substantial amounts reach the intestine and undergo microbiota-dependent biotransformation. This review examines whether parent catechins and their microbial metabolites could modify intestinal processes relevant to α-synuclein misfolding and seeding. Evidence indicates that catechins are converted into phenyl-γ-valerolactones and lower-molecular-weight phenolic metabolites, while microbial amyloids such as curli can promote α-synuclein aggregation in experimental models. EGCG can also remodel α-synuclein assemblies, and catechin exposure can alter microbial and inflammatory phenotypes. However, direct evidence that catechin-derived microbial metabolites inhibit curli–α-synuclein cross-seeding or reduce enteric α-synuclein seeding remains limited. By distinguishing general neuroprotection from effects on aggregation, seeding, and disease modification, this review integrates current evidence and highlights the potential relevance of microbiota-dependent catechin metabolism to gut-origin α-synuclein pathology and body-first PD.

## 1. Introduction

Parkinson’s disease (PD) is a clinically and biologically heterogeneous neurodegenerative disorder characterized by degeneration of nigrostriatal dopaminergic neurons and the accumulation of α-synuclein-rich Lewy pathology [1]. α-Synuclein is an abundant presynaptic protein involved in synaptic-vesicle homeostasis and SNARE-complex assembly [2]. Under pathological conditions, α-synuclein can misfold and assemble into oligomers and fibrils, some of which act as seeds that template the misfolding and recruitment of soluble α-synuclein [3]. Although these processes are interconnected, general neuroprotection, inhibition of α-synuclein aggregation, reduction in seeding activity, and disease modification represent distinct experimental and clinical outcomes.

One proposed trajectory of PD involves the initial development of α-synuclein pathology in peripheral autonomic or enteric structures, followed by centripetal spread to the central nervous system. Here, “gut origin” refers to the proposed formation of pathological α-synuclein in the intestine and its subsequent spread toward the brain. Detecting intestinal α-synuclein, demonstrating its aggregation or seeding activity, and establishing gut-to-brain propagation are distinct levels of evidence. Intestinal detection alone does not establish where pathological α-synuclein originated or whether it subsequently propagated to the brain. In mice, injection of α-synuclein preformed fibrils into the pyloric and duodenal muscularis produced sequential, vagus-dependent propagation of phosphorylated α-synuclein pathology to the brain, followed by neurodegeneration and behavioral deficits [4]. This finding supports the biological plausibility of gut-to-brain propagation but does not establish that PD generally begins in the gut. The brain-first versus body-first framework instead proposes heterogeneous anatomical trajectories, with gut-origin pathology being most relevant to the proposed body-first subtype [5,6]. Human observations of prodromal gastrointestinal dysfunction, intestinal barrier abnormalities, peripheral α-synuclein pathology, and microbiome differences provide additional support for a gut-related component of PD [7,8]. Recent studies have detected α-synuclein seeding activity in duodenal and gastroduodenal biopsies from patients with PD, although detection varies with sampling site and disease stage [9,10]. Chandra et al. reported transfer of α-synuclein from gut mucosal cells to vagal neurons and evidence of seeding activity in the hindbrain [11]. However, these associations remain insufficient to establish the direction or causality of the relationships among dysbiosis, intestinal inflammation, α-synuclein misfolding, and disease progression.

Tea catechins are relevant to this framework through both direct and microbiota-dependent pathways. Among the major tea catechins, epigallocatechin-3-gallate (EGCG) has been shown to redirect α-synuclein assembly and remodel mature fibrils into less toxic, off-pathway structures in biochemical and cellular systems [12,13]. In vivo studies have also reported beneficial effects of green tea catechins on α-synuclein-related and neurobehavioral outcomes, including improved motor performance and reduced circulating α-synuclein in an A53T transgenic mouse model [14]. Together, these findings provide a basis for considering whether catechin-related effects on α-synuclein may also operate within the intestinal environment relevant to gut-origin pathology.

A substantial proportion of orally consumed catechins escapes absorption in the upper gastrointestinal tract and consequently reaches the colon, where it becomes available for microbiota-dependent biotransformation [15]. Colonic metabolism generates phenyl-γ-valerolactones and lower-molecular-weight phenolic acids that contribute substantially to systemic exposure and may have biological properties distinct from those of the parent catechins [16]. At the same time, sustained green tea polyphenol administration can modify microbial community structure and functional potential, demonstrating that the relationship between tea catechins and the gut microbiome is bidirectional [17]. Together, these observations broaden the mechanistic focus from parent catechins alone to the combined effects of parent compounds, microbiota-derived metabolites, and microbial functions that shape the intestinal environment.

A particularly relevant microbial function is the production of extracellular functional amyloids such as curli. In a mouse model, exposure to curli-producing bacteria promoted α-synuclein aggregation and motor impairment, whereas genetic disruption of curli production attenuated these effects [18]. This evidence raises the possibility that microbial amyloid–α-synuclein interactions contribute to enteric seed formation. Microbial inflammatory products, intestinal barrier disruption, oxidative stress, and impaired proteostasis may provide additional conditions that favor α-synuclein misfolding. Within this framework, an important question is whether tea catechins or specific catechin-derived microbial metabolites can influence curli production, curli–α-synuclein cross-seeding, or the formation of seed-competent α-synuclein in the intestine.

Accordingly, this review examines how tea catechins may modulate gut-origin α-synuclein seed formation through the direct actions of parent catechins, microbial biotransformation, and changes in microbial amyloids, intestinal inflammation, barrier integrity, oxidative stress, and proteostasis. Particular attention is given to the potential interactions among tea catechins, microbiota-derived metabolites, curli-mediated cross-seeding, and enteric α-synuclein pathology. Relevant human, animal, cellular, and biochemical studies were identified through narrative Google Scholar searches using combinations of “tea catechins,” “microbial metabolites,” “curli,” “α-synuclein seeding,” and “Parkinson’s disease.” The literature available through August 2026 was considered; no systematic search or formal quality scoring was performed. Throughout the review, evidence is organized according to whether it directly supports these mechanisms, provides indirect or supportive findings, or represents a proposed mechanistic link. By integrating evidence across these levels, this review provides a framework for evaluating the potential of tea catechins and their microbial metabolites to modulate gut-origin α-synuclein seeding and its relevance to PD progression. Studies directly addressing microbial amyloids, catechin metabolism, α-synuclein pathology, or the gut–brain pathway were prioritized.

## 2. Gut-Origin α-Synuclein Seeding as a Potentially Modifiable Target

Constipation and reduced bowel-movement frequency can precede the motor diagnosis of Parkinson’s disease (PD), but these features are nonspecific and do not demonstrate that PD begins in the gut [19]. In the brain-first versus body-first framework, early peripheral autonomic or enteric involvement has been proposed primarily for the body-first trajectory, whereas other cases may develop initially within the central nervous system [5,6]. Enteric α-synuclein seeding should therefore be considered a plausible, phenotype-dependent route in a subset of patients, rather than a universal initiating event in PD.

Human tissue studies provide suggestive but inconsistent support for enteric α-synuclein pathology before PD diagnosis. Phosphorylated α-synuclein immunoreactivity was detected more often in archived gastrointestinal tissues from individuals who were subsequently diagnosed with PD than in control tissues, including samples obtained during the prodromal period [20,21]. However, another study detected aggregated and Ser129-phosphorylated α-synuclein in colonic mucosal biopsies from both participants with PD and controls, demonstrating limited diagnostic specificity [22]. Differences in sampling site and depth, antibodies, tissue processing, pathological criteria, and retrospective study design complicate comparisons and preclude firm conclusions about where or when pathological seeding begins [23].

Experimental inoculation studies support the biological feasibility of enteric seeding and vagal propagation. Injection of α-synuclein preformed fibrils into the mouse gastric wall produced phosphorylated α-synuclein aggregates in the dorsal motor nucleus of the vagus; vagotomy prevented this response, although pathology did not progress beyond the brainstem during the observation period [24]. In a separate model, fibril injection into the pyloric and duodenal muscularis produced sequential, vagus-dependent brain pathology, neurodegeneration, and behavioral deficits [4]. Duodenal fibril inoculation also produced age-dependent outcomes, with progressive brain pathology and motor deficits occurring in aged but not young adult mice [25]. Together, these studies demonstrate that enteric α-synuclein pathology can propagate toward the brain under defined experimental conditions, while also showing that the extent of propagation depends on factors such as age and inoculation protocol.

Clinical studies have identified PD-associated differences in gut microbial composition and correlations among intestinal permeability, mucosal α-synuclein staining, and markers of endotoxin exposure [7,8,26]. Menozzi et al. reported PD-like microbial patterns in some nonmanifesting GBA1 variant carriers in a cross-sectional metagenomic study [27]. These observations support a role for the intestinal environment in PD-associated pathology, although they do not determine whether dysbiosis or barrier dysfunction is a cause, consequence, or modifier of disease [28]. PD-associated constipation and altered bowel transit may themselves influence microbial composition [29]. Medication use, diet, bowel transit, disease stage, and other confounders further complicate interpretation [30]. These observations provide a basis for considering how microbial amyloids and inflammatory bacterial products may influence α-synuclein pathology [18,31].

The gut is also a potentially modifiable site for dietary intervention. A substantial fraction of ingested tea flavan-3-ols reaches the distal intestine, where microbial biotransformation generates phenyl-γ-valerolactones and smaller phenolic metabolites [15,16]. This creates sustained local exposure to both parent catechins and microbiota-derived metabolites within the same intestinal environment in which microbial amyloids, inflammatory signals, and α-synuclein pathology may interact. These features provide a mechanistic basis for considering tea catechins as potential modulators of enteric α-synuclein seeding and propagation, particularly in the context of body-first PD.

## 3. Microbial Amyloids and Inflammatory Products in Enteric α-Synuclein Pathology

Microbial amyloids and inflammatory bacterial products represent distinct but potentially convergent influences on gut-origin α-synuclein pathology. The strongest experimental evidence concerns curli, an extracellular functional amyloid assembled from CsgA and CsgB subunits. Studies using purified-protein assays, bacterial genetics, *Caenorhabditis elegans*, and mammalian models collectively support the ability of selected curli systems to promote α-synuclein aggregation and related pathological phenotypes under defined experimental conditions [18,31,32,33]. These models measure different endpoints and should be considered as different levels of evidence.

Direct evidence for cross-seeding comes primarily from controlled biochemical and cellular systems. Purified *Escherichia coli* CsgA accelerated α-synuclein fibril formation in vitro, and aggregates generated in the presence of CsgA retained seeding activity [18]. CsgA homologues encoded by bacteria represented in the human gut microbiome differed in their effects on α-synuclein aggregation; slowly aggregating homologues formed mixed complexes with α-synuclein that provided nuclei for faster fibril growth [33]. Biofilm-associated protein (BAP) amyloids also accelerated α-synuclein fibrillization in cell-free assays and increased α-synuclein aggregation in neuronal cells [34]. Together, these studies provide direct biochemical evidence that bacterial amyloids can alter α-synuclein assembly and seeding behavior. These assay results do not, by themselves, demonstrate gut-to-brain propagation or Parkinson’s disease in humans.

Genetic experiments provide an additional level of causal evidence. In a genome-wide *Escherichia coli* knockout screen using *C. elegans* models expressing human α-synuclein, deletion of *csgA* or *csgB* reduced α-synuclein-associated neuronal death and improved mitochondrial and neuronal phenotypes [31]. In α-synuclein-overexpressing mice, colonization with curli-producing *E. coli* worsened intestinal and brain α-synuclein pathology and motor and gastrointestinal deficits relative to a curli-deficient isogenic strain [18]. These findings support a curli-dependent contribution to α-synuclein-related phenotypes across complementary experimental systems.

Inflammatory bacterial products should be distinguished mechanistically from direct amyloid cross-seeding [35]. In aged rats, curli exposure was associated with increased α-synuclein deposition, gliosis, and elevated innate-immune markers [32,36]. The bacterial knockout screen also identified lipopolysaccharide-assembly genes, including *lapA* and *lapB*, whose disruption reduced α-synuclein-associated neurodegeneration in *C. elegans* [31]. These findings indicate that bacterial inflammatory pathways can influence host α-synuclein-related phenotypes independently of direct amyloid cross-seeding. Curli can induce enteric inflammatory responses, but whether these responses increase seed-competent α-synuclein remains unresolved [36]. Intestinal inflammation, oxidative stress, barrier dysfunction, and impaired proteostasis may therefore act as complementary mechanisms that create an environment favorable to α-synuclein misfolding and aggregation.

Table 1 organizes these studies according to evidence type, distinguishing direct biochemical cross-seeding, bacterial genetic causality, in vivo promotion, inflammatory associations, and unresolved mechanisms. This distinction is particularly relevant to the tea-catechin framework. EGCG inhibited CsgA-accelerated α-synuclein aggregation in vitro and reduced curli-associated phenotypes in a mouse model [18], linking catechin activity to one of the best-characterized microbial amyloid pathways associated with α-synuclein pathology.

## 4. Tea Catechins: Beyond Classical Antioxidant Neuroprotection

The microbial-amyloid evidence described above provides a rationale for examining whether parent tea catechins can influence α-synuclein-related processes. Among the major tea catechins, (−)-epigallocatechin-3-gallate (EGCG) has received the greatest attention because of its reported effects on α-synuclein assembly, oxidative stress, inflammatory signaling, and proteostasis. Evidence from cell-free, cellular, and animal models suggests that these effects occur at multiple biological levels and should be interpreted according to the specific endpoint measured.

Cell-free studies show that EGCG can alter α-synuclein assembly under defined experimental conditions. EGCG redirected α-synuclein away from β-sheet-rich fibrils toward unstructured off-pathway oligomers in purified-protein assays [12] and remodeled mature α-synuclein fibrils into smaller amorphous aggregates with reduced toxicity in a mammalian-cell assay [13]. Xu et al. also reported concentration-dependent inhibition of recombinant α-synuclein fibril formation and reduced binding of a fluorescent α-synuclein probe to aggregates in postmortem PD tissue [38]. Together, these findings support direct effects of EGCG on α-synuclein aggregation and fibril structure.

The cell-free literature also demonstrates that these effects are condition-dependent. Sternke-Hoffmann et al. found that EGCG inhibition varied with solution conditions and EGCG oxidation; under some conditions, EGCG accelerated fibril formation, and quantitative seeding experiments showed that remodeled fibrils retained seeding competence [39]. These findings emphasize an important distinction: inhibition of aggregation, fibril remodeling, and loss of seeding activity are related but not interchangeable outcomes. Consequently, remodeling α-synuclein fibrils cannot be assumed to eliminate their seed competence.

Cellular experiments provide a separate level of evidence. In α-synuclein-overexpressing PC12 cells, Zhao et al. reported reduced intracellular α-synuclein fibrillation and reactive oxygen species together with improved cell viability after EGCG treatment [40]. Reduced reactive oxygen species or improved cell viability alone should not be interpreted as evidence of reduced α-synuclein seeding. Such findings support a broader neuroprotective role for EGCG that may involve antioxidant, anti-inflammatory, mitochondrial, autophagic, lysosomal, or proteostasis-related mechanisms in addition to direct effects on α-synuclein assembly.

Animal studies further extend these observations. In α-synuclein-overexpressing mice colonized with curli-producing *Escherichia coli*, oral EGCG reduced *csgA* expression and curli-associated biofilm formation, inhibited CsgA-accelerated α-synuclein aggregation in vitro, and improved gastrointestinal, motor, inflammatory, and brain α-synuclein outcomes [18]. This study is particularly relevant because it connects a tea catechin with both a bacterial amyloid pathway and host α-synuclein pathology. In a separate brain-directed model, Shen et al. administered EGCG before intrastriatal α-synuclein preformed-fibril inoculation and reported improved behavioral outcomes, preservation of tyrosine hydroxylase-positive neurons, reduced phosphorylated α-synuclein pathology, and altered inflammatory markers [41]. Although this model bypasses the gastrointestinal tract, it provides complementary evidence that EGCG can influence α-synuclein-related pathology in vivo. This brain-directed model therefore cannot directly test prevention of gut-origin α-synuclein seed formation.

In a study from our laboratory, Riegelman et al. evaluated green tea polyphenols in 30 six-month-old male hemizygous A53T α-synuclein-transgenic mice assigned to 0%, 0.5%, or 1.5% treatment in drinking water for 90 days [14]. Catechin exposure was confirmed by urinary metabolite analysis, while open-field and rotarod performance were monitored longitudinally, and plasma α-synuclein was measured at the end of treatment. Green tea polyphenol treatment maintained or improved rotarod performance as control performance declined, and the high-dose group showed lower plasma α-synuclein than controls. These findings provide in vivo evidence for beneficial neurobehavioral and peripheral α-synuclein-related effects of sustained green tea polyphenol exposure [14]. Because this intervention was a polyphenol mixture, these outcomes cannot be attributed to EGCG alone. One study by Xu et al. has reported improved behavioral outcomes and neuronal health in *Drosophila* model following a treatment with EGCG with effects involving TotM and gut microbiota [42]. These finding shows microbiota dependent neuroprotection but do not show inhibition in the seeding of enteric α-synuclein. 

The biological relevance of parent catechins must also be considered in the context of their pharmacokinetics. Parent catechins have limited and variable systemic bioavailability, whereas substantial gastrointestinal exposure and extensive microbial conversion occur after ingestion [43,44]. In a human study using 1.5–4.5 g of decaffeinated green tea solids, peak plasma EGCG reached 326 ng/mL (approximately 0.71 µM); this measurement does not establish intestinal tissue concentrations [45]. This distribution limits direct extrapolation from purified-protein and cell systems to the brain but increases the potential importance of intestinal exposure, microbial biotransformation, and locally generated metabolites. Parent-catechin effects and microbiota-dependent effects should therefore be evaluated separately; defined microbial metabolites have not yet been shown to inhibit enteric α-synuclein seeding directly.

Table 2 summarizes the evidence for parent catechins and catechin-derived metabolites across experimental levels, distinguishing aggregation, cellular, behavioral, pathological, and biomarker outcomes from evidence directly related to enteric α-synuclein seeding and propagation.

## 5. Microbial Biotransformation of Tea Catechins: Established Metabolism and Candidate Bioactivities

The microbial biotransformation of tea catechins begins with their incomplete absorption in the upper gastrointestinal tract. The major parent flavan-3-ols—epicatechin (EC), epigallocatechin (EGC), epicatechin gallate (ECG), and epigallocatechin-3-gallate (EGCG)—differ in absorption and metabolic fate. Absorbed catechins undergo extensive host phase II conjugation, whereas a substantial unabsorbed fraction reaches the distal intestine and becomes available for microbial metabolism [48,49]. In an ex vivo human ileostomy-fluid model, cleavage of the gallic acid ester was observed for the gallated catechins EGCG and ECG, and phenyl-γ-valerolactones were detected after incubation with catechins [50]. These findings support degalloylation as an early metabolic step for gallated catechins, but not for non-gallated catechins.

Microbial C-ring opening and fission generate phenyl-γ-valerolactones and phenylvaleric acids, followed by further catabolism to lower-molecular-weight phenylpropionic, phenylacetic, benzoic, and related phenolic acids [46,51]. In isolated human intestinal bacteria, *Eggerthella lenta* rK3 cleaved the C-ring of epicatechin and catechin, while *Flavonifractor plautii* aK2 converted the resulting intermediate into a phenyl-γ-valerolactone and a phenylvaleric acid [52]. These microbial products are distinct from the glucuronide, sulfate, and methyl conjugates subsequently generated by host enzymes. Human metabolomics after one year of green tea polyphenol supplementation identified fecal and urinary valerolactones and phenylvaleric acid products while showing relatively limited changes in overall fecal microbial community composition [53]. Urinary phenyl-γ-valerolactones can therefore serve as biomarkers of flavan-3-ol exposure, although they are not specific to tea because other flavan-3-ol-rich foods generate the same metabolite class [54].

The biological activities of these microbial metabolites represent a distinct level of evidence from their formation and systemic exposure. Their mechanistic relevance is supported by three features: they can contribute substantially to circulating flavan-3-ol-derived metabolites, their production depends on gut microbial biotransformation, and their biological activities may differ from those of the parent catechins. In LPS- or tert-butyl hydroperoxide-stimulated SH-SY5Y cells and LPS-stimulated RAW 264.7 macrophages, selected microbial phenolic acids and conjugates altered cell viability, reactive oxygen species, and cytokine release [47]. These findings support cytoprotective and anti-inflammatory activities of selected catechin-derived metabolites and suggest that microbial metabolism may extend the biological effects of tea catechins beyond those of the parent compounds.

**Figure 1:**
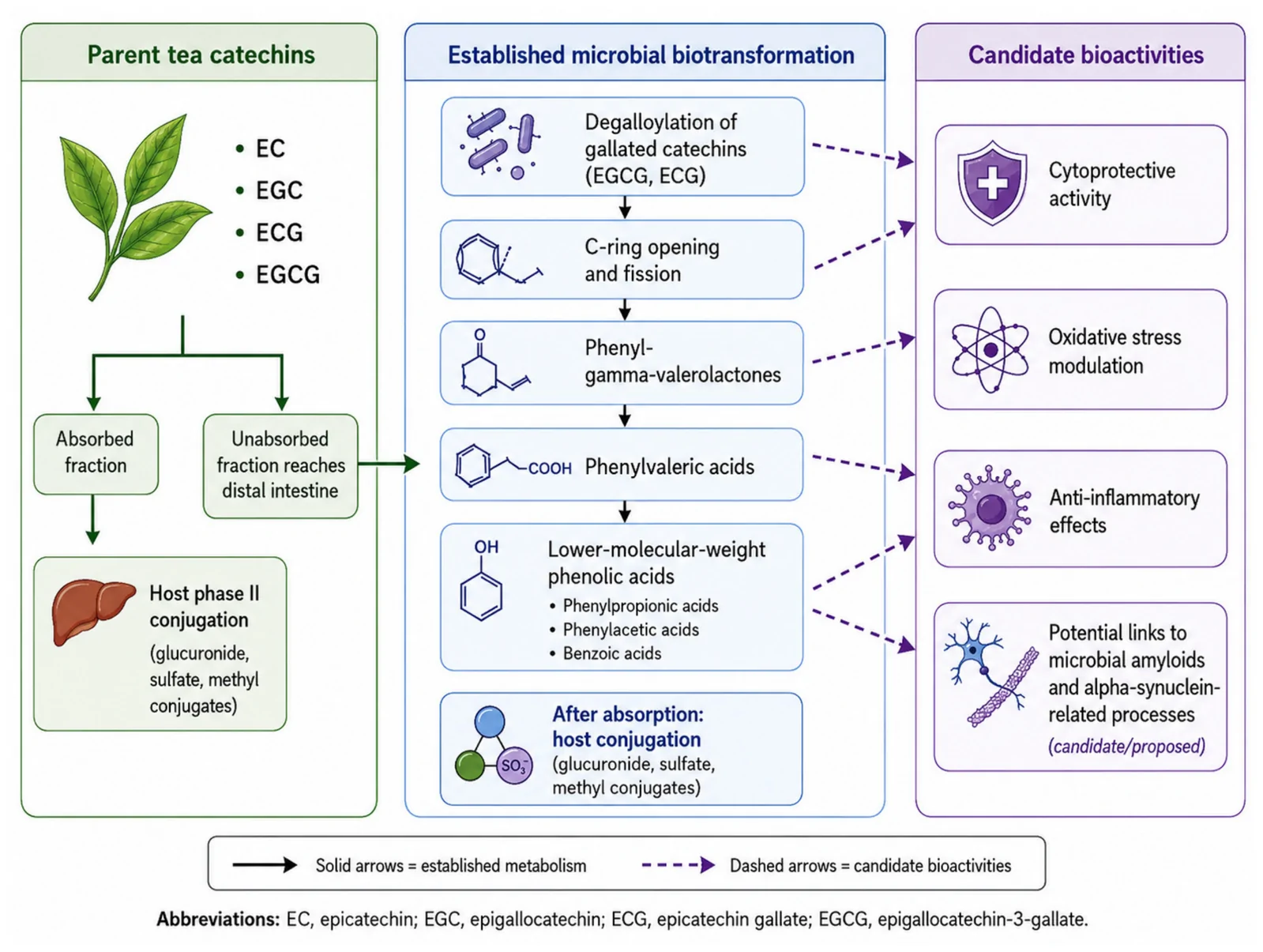
Established microbial biotransformation of tea catechins and candidate bioactivities of the resulting metabolites.

Interindividual variation in urinary valerolactone output after green tea exposure and in vitro fecal incubation experiments have indicated substantial variability in catechin bioavailability and microbialmetabolism [44,55]. Such variation may contribute to differences in biological responses to tea catechin intake by altering the profile and abundance of metabolites generated within the gut and reaching the systemic circulation. Background diet, bowel function, PD medication use, and disease stage should be considered when comparing microbial and metabolite profiles across individuals [56]. Broader interactions among dietary polyphenols, the gut microbiota, and α-synuclein-related pathways have been reviewed previously [57]. In the present review, emphasis is placed on catechin-derived microbial metabolites as potential links between intestinal catechin metabolism, microbial amyloids, and α-synuclein-related processes. Figure 1 summarizes the established metabolic transformations and the candidate bioactivities of the resulting metabolites.

## 6. Experimental Platforms for Testing the Proposed Gut-Centered Framework

Different experimental platforms capture distinct aspects of α-synuclein biology, including aggregation, seeding activity, cellular protection, enteric pathology, and gut-to-brain propagation. Cell-free thioflavin-T kinetics, sedimentation or filter-trap assays, electron microscopy, and matched seeding assays can distinguish changes in fibril formation from changes in seed competence. Purified CsgA or curli fibrils can be combined with α-synuclein to examine heterologous cross-seeding directly, while accounting for assay conditions and catechin oxidation state [33,39].

Cellular systems provide complementary mechanistic information. SH-SY5Y and PC12 models can assess viability, intracellular fibrillation, reactive oxygen species, and inflammatory responses, whereas induced pluripotent stem cell-derived neurons offer greater human neuronal relevance. FRET biosensor cells quantify seeded intracellular aggregation [58], while RT-QuIC represents a distinct seed-amplification approach and should be interpreted separately [9]. Intestinal epithelial cells, intestinal organoids, and enteric neuronal systems extend these approaches to barrier function, epithelial responses, neuroglial interactions, and seed-transfer processes, although they do not reproduce the full microbial, immune, and vagal environment.

Genetically matched curli-producing and curli-deficient bacteria provide a direct means of isolating the contribution of microbial amyloids. These models can distinguish nonspecific antimicrobial effects from changes in CsgA expression, curli abundance or assembly, biofilm phenotypes, and catechin-metabolite production, thereby helping to define whether observed effects are specifically linked to microbial amyloid pathways.

*C. elegans* provides a tractable in vivo system in which bacterial exposure can be tightly controlled while multiple organism-level outcomes are measured. NL5901 expresses human α-synuclein::YFP in body-wall muscle and is primarily suited to quantifying α-synuclein accumulation [59]. UA44 expresses human α-synuclein and GFP in dopaminergic neurons and can be used to assess age-dependent dopaminergic-neuron integrity and dopamine-related behavior [60]. BZ555 expresses GFP in dopaminergic neurons without human α-synuclein expression and therefore serves as a reporter of dopaminergic-neuron integrity [61]. CeMbio enables strain- and community-level perturbation within a defined worm-associated microbiota, although its correspondence to the human gut microbiome is necessarily limited [62].

Rodent models address complementary levels of biological complexity. Transgenic α-synuclein models capture chronic host susceptibility, while exposure to curli-producing bacteria examines microbial-amyloid effects in vivo. Gastrointestinal PFF inoculation followed by assessment of vagus-dependent propagation provides a model of anatomically defined gut-to-brain spread [4], whereas brain-directed or systemic PFF paradigms examine centrally initiated α-synuclein pathology. The intrastriatal EGCG study and the A53T green-tea-polyphenol study provide evidence for brain-directed pathological effects and behavioral or peripheral-biomarker outcomes, respectively [14,41].

Human studies currently provide the strongest translational context for exposure, biomarker, and disease-association questions. Gastrointestinal α-synuclein seeding activity can be assessed using seed-amplification assays [9,10,63], while urinary phenyl-γ-valerolactones provide biomarkers of flavan-3-ol exposure [54]. Integration of dietary exposure, circulating or urinary metabolites, stool metagenomics and metabolomics, gastrointestinal α-synuclein seeding activity, medication use, bowel function, and longitudinal clinical outcomes may help clarify how microbiota-dependent catechin metabolism relates to body-first PD phenotypes. Table 3 summarizes the principal readouts, strengths, and limitations of these complementary platforms.

## 7. Mechanistic Integration and Future Priorities

Current evidence can be organized into three complementary domains. First, EGCG can alter α-synuclein aggregation or fibril structure in defined cell-free and cellular systems, although the magnitude and direction of these effects depend on experimental conditions [13,39]. Second, intestinal microbes transform parent catechins into phenyl-γ-valerolactones, phenylvaleric acids, and lower-molecular-weight phenolic products [46,50]. Third, microbial amyloids such as curli can promote α-synuclein aggregation and PD-related phenotypes in experimental systems [18,31,32,33]. Together, these domains establish a mechanistic basis for considering a connected catechin–microbiome–α-synuclein pathway while keeping the strength of evidence for each link distinct.

Additional supportive evidence comes from catechin-associated changes in oxidative stress, inflammatory signaling, barrier-related endpoints, cellular viability, microbial composition, and bacterial biofilm phenotypes. These effects may influence the intestinal environment in which α-synuclein misfolding and aggregation occur, but they are mechanistically distinct from direct effects on seed competence or microbial amyloid–α-synuclein cross-seeding.

**Figure 2:**
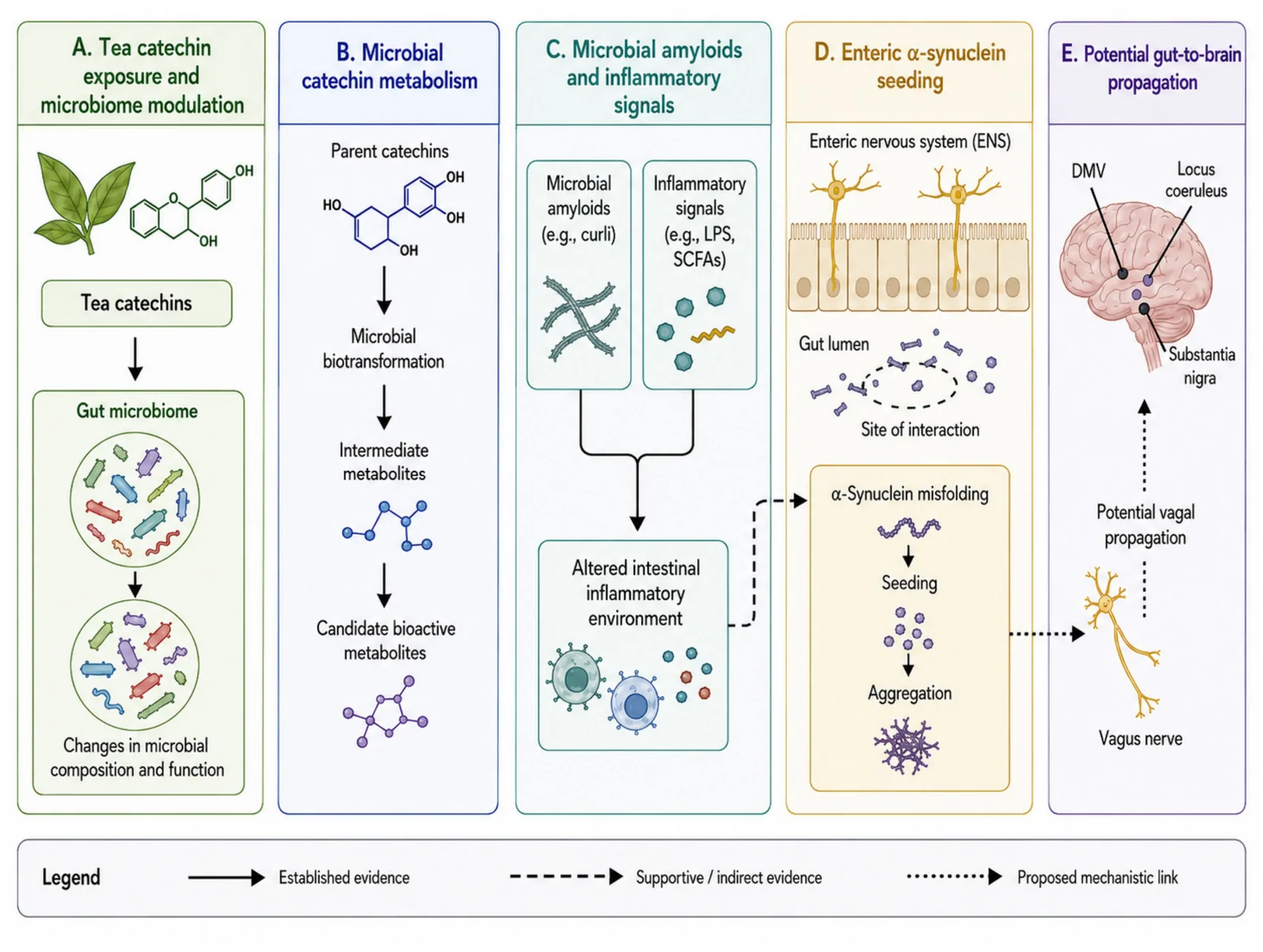
Integrative framework linking tea catechin exposure, microbial metabolism, microbial amyloids and inflammatory signals, enteric α-synuclein seeding, and potential gut-to-brain propagation. Solid arrows indicate relationships supported by established experimental evidence, dashed arrows indicate supportive or indirect evidence, and dotted arrows indicate proposed mechanistic links.

As summarized in Figure 2, the major unresolved links are those connecting catechin exposure and microbial metabolism to enteric α-synuclein seeding. These include the extent to which parent catechins or defined microbial metabolites alter CsgA expression or curli abundance, modify curli–α-synuclein cross-seeding, influence seed-competent α-synuclein in intestinal tissue, and affect subsequent anatomically connected pathology. Clarifying these relationships will require experimental designs that distinguish general antimicrobial, cytoprotective, and anti-inflammatory effects from specific actions on microbial amyloid production and α-synuclein seeding.

Future work will benefit from integrating evidence across experimental levels. Cell-free systems can define effects on aggregation, fibril structure, cross-seeding, and seed competence; bacterial genetic models can establish curli dependence; intestinal cellular and organoid systems can assess epithelial, inflammatory, and seed-transfer responses; and in vivo models can connect intestinal seeding with enteric, vagal, and brain pathology. Matched experiments should compare parent catechins, defined microbial metabolites, and their combination for fibrillization, oligomerization, seed activity, and cell-to-cell transfer, while identifying the microbial genes and enzymes responsible for differences in metabolite production. Human studies can then provide translational context by relating catechin exposure, microbial metabolite profiles, microbiome features, gastrointestinal α-synuclein seeding activity, and clinical phenotypes. Longitudinal cohorts spanning at-risk, prodromal, and established PD should integrate metagenomics, metabolomics, proteomics, and α-synuclein seeding assays. Such integration is necessary to determine whether microbiota-dependent catechin metabolism modifies α-synuclein seeding itself or instead influences broader neuroprotective pathways associated with PD. Before claiming PD prevention, human intervention studies should establish the sequence from measured exposure and metabolites to a biological target, biomarker, and clinical outcome.

## 8. Limitations of the Current Evidence and Review

Direct studies testing whether catechin-derived metabolites inhibit curli-mediated α-synuclein seeding are lacking. The studies included in this review use heterogeneous models, exposures, and endpoints, which limit comparisons and extrapolation to concentrations attainable through tea consumption. Human longitudinal data linking catechin metabolism to gastrointestinal α-synuclein seed activity and PD outcomes are unavailable, and distinguishing a body-first trajectory from other PD subtypes remains difficult. Diet, constipation, medication use, and disease stage can confound microbiome associations. Finally, this narrative review did not use systematic study selection or formal quality scoring; its evidence categories are qualitative judgments.

## 9. Conclusions

Tea catechins may influence multiple processes relevant to Parkinson’s disease. EGCG can modify α-synuclein aggregation, intestinal microbes convert catechins into metabolites, and microbial amyloids can promote α-synuclein aggregation and related pathology in experimental models. Together, these findings motivate a possible link between catechin exposure, microbial metabolism, and gut-origin α-synuclein pathology. However, current evidence does not establish that tea catechins or their microbial metabolites prevent or modify Parkinson’s disease by inhibiting gut-origin α-synuclein seeding.

Importantly, neuroprotection, anti-inflammatory effects, inhibition of microbial amyloids, suppression of cross-seeding, and reduction in α-synuclein seed activity are distinct outcomes. Integrating biochemical, bacterial, cellular, *C. elegans*, rodent, and human evidence will be necessary to clarify how these processes are connected.

The testable hypothesis is that microbiota-dependent catechin metabolism may alter enteric α-synuclein seed activity relevant to the proposed body-first subtype of PD; direct experiments and longitudinal human studies are needed to evaluate it.

## Figures and Tables

**Table 1:** Synthesis of published evidence linking microbial amyloids and inflammatory bacterial pathways to α-synuclein aggregation and PD-related phenotypes, organized by evidence type.

Microbial Product	Source/Control	Model	Measured Endpoints	Finding	Evidence Level	Citation
Curli (CsgA)	Curli-producing *E. coli* and matched curli-deficient control	Rats and α-synuclein-expressing *C. elegans*	Gut/brain α-synuclein deposition; glial and inflammatory markers; worm aggregates	Curli-producing exposure increased α-synuclein-related outcomes relative to controls.	Curli-dependent in vivo promotion; not direct biochemical cross-seeding	[32]
Curli/CsgA	Curli-producing and curli-deficient *E. coli*; purified CsgA	α-synuclein-overexpressing mice plus cell-free assay	Gut/brain pathology, GI/motor outcomes, aggregation kinetics	Curli expression promoted pathology and purified CsgA accelerated aggregation.	Parallel in vivo causal and direct biochemical evidence	[18]
Curli pathway	*E. coli* single-gene deletion library	Dopaminergic α-synuclein *C. elegans* model	Neuronal survival, mitochondrial function, α-synuclein toxicity	Curli-pathway deletion reduced α-synuclein-associated neurodegeneration.	Causal bacterial genetic evidence	[31]
CsgA homologues	Human-microbiome-associated bacterial CsgA homologues	Cell-free biophysical assays	Aggregation kinetics and complex formation	Homologues differed in their ability to accelerate α-synuclein aggregation.	Direct biochemical cross-seeding	[33]
*Desulfovibrio* spp.	PD-patient and healthy-spouse isolates	NL5901 *C. elegans* feeding model	Aggregate number/size and survival	Patient-derived isolates produced more α-synuclein aggregates than spouse-derived isolates.	In vivo association; mechanism unresolved	[37]
Biofilm-associated protein amyloids	Human gut commensals and BAP genes	Purified amyloid, *C. elegans*, mouse brain inoculation, metagenomics	α-Synuclein aggregation, pathology, BAP-gene abundance	BAP amyloid promoted α-synuclein-related phenotypes across experimental systems.	Experimental promotion plus human metagenomic association	[34]
LPS biosynthesis pathway	Gram-negative bacterial gene deletions	*C. elegans*	Host neurodegeneration	Deletion of bacterial LPS-biosynthesis genes reduced α-synuclein-associated neurodegeneration.	Causal bacterial genetic evidence; not cross-seeding	[31]

Abbreviations: α-syn, α-synuclein; BAP, biofilm-associated protein; CsgA, curli subunit proteins; LPS, lipopolysaccharide; PD, Parkinson’s disease.

**Table 2:** Synthesis of published evidence for tea catechins and catechin-derived metabolites in α-synuclein-related models, organized by experimental level and relevance to gut-origin pathology.

Compound/Metabolite	Model	Measured Endpoints	Finding	Gut-Origin Relevance	Evidence Level	Citation
EGCG	Recombinant fibrils and mammalian-cell toxicity assay	Fibril remodeling and toxicity	Mature fibrils were remodeled into smaller amorphous aggregates with reduced toxicity.	Direct effects on α-synuclein assembly; gut-origin relevance indirect	Cell-free/cellular	[13]
EGCG	Recombinant α-synuclein; postmortem PD tissue probe	Fibril formation and probe binding	Concentration-dependent aggregation inhibition and reduced probe binding.	Direct aggregation evidence; gut-origin relevance indirect	Cell-free/tissue-binding	[38]
EGCG (counterevidence)	α-Synuclein aggregation and quantitative seeding assays	Fibril kinetics and seeding competence	Effects depended on conditions; EGCG could accelerate aggregation and did not abolish seeding competence.	Directly cautions against equating remodeling with seed loss	Cell-free seeding	[39]
EGCG	α-Synuclein-overexpressing PC12 cells	Fibrillation, ROS, viability	Reduced intracellular fibrillation and ROS with improved viability.	Cellular neuroprotection; gut-origin relevance indirect	Cellular	[40]
EGCG	Intrastriatal PFF mouse model	Behavior, TH-positive neurons, pSer129 α-synuclein, cytokines	Pretreatment improved selected behavioral and pathological outcomes.	Brain-directed model bypassed the gut	Rodent, centrally seeded	[41]
Green tea polyphenols	Hemizygous A53T α-synuclein-transgenic mice; 0%, 0.5%, or 1.5% in drinking water for 90 days	Open-field and rotarod performance; plasma α-synuclein; urinary catechins/metabolites	Treatment preserved or improved rotarod performance, and high-dose treatment reduced plasma α-synuclein.	Demonstrates sustained catechin exposure and α-synuclein-related in vivo effects; gut-origin mechanism not assessed	Transgenic rodent behavioral/biomarker evidence	[14]
Phenyl-γ-valerolactones/phenylvaleric acids	Human flavan-3-ol metabolism literature	Formation, bioavailability, variability	Major microbial flavan-3-ol metabolites with variable production.	Establishes microbial metabolite exposure relevant to intestinal and systemic effects	Human pharmacokinetic/metabolite evidence	[46]
Microbial phenolic acids/conjugates	Stimulated SH-SY5Y and RAW 264.7 cells	Viability, ROS, cytokines	Selected metabolites altered cytoprotective and inflammatory endpoints.	Supports biological activity of microbial metabolites; α-synuclein relevance indirect	Cellular mechanistic evidence, indirect to α-synuclein	[47]

Abbreviations: EGCG, epigallocatechin-3-gallate; PD, Parkinson’s disease.

**Table 3:** Evidence-informed comparison of experimental platforms for evaluating the proposed tea catechin–microbiome–α-synuclein framework.

Category	Platform/Model	Primary Question	Key Readouts	Strengths	Limitations	Citations
Cell-free/biochemical	Aggregation and cross-seeding assays	Does a compound alter α-synuclein aggregation, cross-seeding, or seed competence?	ThT kinetics; EM; sedimentation/filter-trap; normalized seeding assays	High mechanistic control; direct assessment of protein interactions	Strongly condition-dependent; lacks host context	[33,39]
Bacterial	Bacterial endpoint assays	Does exposure specifically alter curli production or assembly?	Growth; Congo red binding; CsgA expression; curli abundance; biofilm formation; metabolites	Direct and scalable	Limited to bacterial phenotypes	[18]
Bacterial	Curli-positive vs. ΔcsgA bacteria	Is bacterial amyloid required for the observed host phenotype?	Matched host phenotypes; α-synuclein outcomes; inflammatory endpoints	Strong bacterial genetic control	Establishes curli dependence but not the molecular route of cross-seeding	[18,32]
Cell-based	Neuronal cells/iPSC-derived neurons	Does exposure alter α-synuclein toxicity or intracellular aggregation?	Viability; ROS; intracellular fibrillation; pSer129 α-synuclein	Scalable; iPSC-derived neurons increase human relevance	Limited microbial, intestinal, and anatomical context	[40]
Seed amplification/biochemical	FRET biosensor cells	Does exposure alter intracellular seeding activity?	FRET-positive cells; integrated FRET density	Sensitive quantitative measure of seeded aggregation	Reporter-based cellular endpoint	[58]
Seed amplification/biochemical	RT-QuIC	Does exposure alter amplifiable α-synuclein seed activity?	Lag time; maximum fluorescence; positive fraction	Highly sensitive assessment of seeding activity	Measures amplifiable seed activity rather than anatomical propagation	[9]
Cell-based/intestinal	Intestinal cells, organoids, and enteric neurons	How does the intestinal environment influence α-synuclein accumulation, barrier responses, or seed transfer?	Barrier integrity; epithelial/neuroglial responses; α-synuclein accumulation; seed activity	Provides gut-specific cellular and tissue context	Incomplete microbial, immune, and vagal complexity	[11,36]
*C. elegans*	NL5901	Does bacterial diet or exposure alter α-synuclein accumulation?	Body-wall-muscle α-synuclein::YFP; motility; survival	Quantifiable in vivo model with controlled bacterial exposure	α-Synuclein is expressed in body-wall muscle rather than dopaminergic neurons	[59]
*C. elegans*	UA44	Does exposure modify α-synuclein-associated dopaminergic-neuron injury?	Dopaminergic-neuron integrity; dopamine-related behavior	Neuron-specific α-synuclein model with controlled bacterial diet	Simplified nervous, immune, and intestinal systems	[60]
*C. elegans*	BZ555	Does exposure preserve dopaminergic-neuron integrity?	dat-1p::GFP neuronal morphology	Direct in vivo reporter of dopaminergic-neuron integrity	Does not express human α-synuclein	[61]
*C. elegans* microbiome	CeMbio	Which bacterial strains or communities modify host phenotypes?	Colonization; community composition; metabolites; host phenotypes	Defined, traceable microbial community	Limited correspondence to the human gut microbiome	[62]
Rodent	Transgenic/curli-exposure rodents	Does microbial amyloid modify α-synuclein-related pathology in a mammalian host?	Intestinal and brain α-synuclein pathology; inflammation; microbiome; GI and motor behavior	Intact mammalian physiology and host–microbiome interactions	Model-dependent effects; microbiome and transgene-related variability	[18]
Rodent	Gastrointestinal PFF inoculation	Can enteric α-synuclein pathology propagate to the brain through the vagal route?	Enteric, vagal, brainstem, and staged brain pathology; vagotomy response	Directly models gut-to-brain propagation	Spread depends on inoculation site, age, dose, and observation period	[4]
Rodent	Brain-directed PFF models	Does a compound modify centrally initiated α-synuclein pathology?	pSer129 α-synuclein; TH-positive neurons; cytokines; behavior	Reproducible model of progressive brain pathology	Bypasses gut-origin initiation	[41]
Human	Clinical and biomarker studies	How are catechin exposure, microbial metabolites, gut microbiome features, and GI α-synuclein seeding related to humans?	Diet; plasma/urinary metabolites; stool omics; GI SAA; longitudinal clinical outcomes	Highest translational relevance	Confounding, interindividual heterogeneity, and limited integrated longitudinal datasets	[9,10,54,63]

## Data Availability

No new data were created or analyzed in this study. Data sharing is not applicable to this article.
